# Inform: Efficient Information-Theoretic Analysis of Collective Behaviors

**DOI:** 10.3389/frobt.2018.00060

**Published:** 2018-06-11

**Authors:** Douglas G. Moore, Gabriele Valentini, Sara I. Walker, Michael Levin

**Affiliations:** ^1^BEYOND: Center for Fundamental Concepts in Science, Arizona Sate University, Tempe, AZ, United States; ^2^Department of Biology, Allen Discovery Center, Tufts University, Medford, MA, United States

**Keywords:** information transfer, information storage, information dynamics, complex systems, collective behavior, information theory

## Abstract

The study of collective behavior has traditionally relied on a variety of different methodological tools ranging from more theoretical methods such as population or game-theoretic models to empirical ones like Monte Carlo or multi-agent simulations. An approach that is increasingly being explored is the use of information theory as a methodological framework to study the flow of information and the statistical properties of collectives of interacting agents. While a few general purpose toolkits exist, most of the existing software for information theoretic analysis of collective systems is limited in scope. We introduce Inform, an open-source framework for efficient information theoretic analysis that exploits the computational power of a C library while simplifying its use through a variety of wrappers for common higher-level scripting languages. We focus on two such wrappers here: PyInform (Python) and rinform (R). Inform and its wrappers are cross-platform and general-purpose. They include classical information-theoretic measures, measures of information dynamics and information-based methods to study the statistical behavior of collective systems, and expose a lower-level API that allow users to construct measures of their own. We describe the architecture of the Inform framework, study its computational efficiency and use it to analyze three different case studies of collective behavior: biochemical information storage in regenerating planaria, nest-site selection in the ant *Temnothorax rugatulus*, and collective decision making in multi-agent simulations.

##  1. Introduction

Collective behaviors, such as the coordinated motion of a flock of starlings (Ballerini et al., 2008), the collective decisions made by bees and ants (Franks et al., 2002), and the coordination of individual cells towards the creation or repair of a complex anatomical structure during embryogenesis or regeneration (Pezzulo and Levin, 2015), are complex collective phenomena that emerge from local interactions between many individuals. The study of these complex phenomena has been approached from many different angles, e.g., population models based on ordinary differential equations to predict the dynamics and study the stability of collective behaviors (Couzin et al., 2005; Marshall et al., 2009); game-theoretic approaches to study the emergence of cooperative strategies (Challet and Zhang, 1997); and multi-agents simulations to explore systems in the detail (Goldstone and Janssen, 2005). Another interesting approach is to focus on the distributed computation performed by the individuals in the collective (Langton, 1990; Mitchell, 1996; Lizier et al., 2014) and use information theory to analyze its architecture. Information theory has been used, for example, to detect leadership relations between zebra fishes (Butail et al., 2016; Mwaffo et al., 2017) or to study foraging behavior of ant colonies (Reznikova and Ryabako, 1994; Zenil et al., 2015; Meyer, 2017). Additionally, it is extensively employed in the study of other complex systems with applications ranging from computational neuroscience (Honey et al., 2007; Vakorin et al., 2009; Lizier et al., 2011; Wibral et al., 2014), collectives of artificial agents (Williams and Beer, 2010; Boedecker et al., 2012; Walker et al., 2013; Biehl et al., 2016), neural and Boolean network models (Lizier et al., 2009; Kim et al., 2015; Walker et al., 2016), and multi-robot systems (Sperati et al., 2008; Sperati et al., 2011). Computing information theoretic measures, however, is computationally demanding and requires efficient software methodologies.

A common approach is to develop software solutions to compute specific information-theoretic measures. For example, TRENTOOL (Lindner et al., 2011) and MuTE (Montalto et al., 2014) are Matlab toolkits to compute transfer entropy. MVGC (Barnett and Seth, 2014) has been developed to compute Granger causality while ACSS (Gauvrit et al., 2016) and OACC (Soler-Toscano et al., 2014) to compute approximations to Kolmogorov complexity. However, while software options can always be developed to focus on particular techniques or methods, this approach is time-consuming for end-users. It can be tedious to explore and analyze the complex behavior of systems if every measure one chooses to use requires a separate library, not to mention the time spent in search of the functionality. What’s more, it is not always easy to find a library to suit one’s needs. One solution is to develop and make use of general-purpose software frameworks which can be applied across domains, and can provide researchers from different disciplines with a common software toolkit. At the risk of overselling our current endeavour, we can liken this approach to the development of solid, powerful linear algebra libraries such as BLAS (Lawson et al., 1979) and LAPACK (Anderson et al., 1999) which provide vast array of features and greatly simplify scientific computation. The most notable effort in this direction is the Java Information Dynamics Toolkit (JIDT) developed by (Lizier, 2014). JIDT is a Java library that provides access to classic information-theoretic measures (e.g., entropy and mutual information) as well as more recent measures of information dynamics (e.g., active information and transfer entropy) for both discrete and continuous data. JIDT is general-purpose and, thanks to the flexibility of the Java Virtual Machine, it can be called from several different high-level languages such as Matlab, Python or R.

In previous work (Moore et al., 2017), we introduced Inform: an open-source, general-purpose and cross-platform framework to perform information-theoretic analysis of collective of agents. Inform is a framework to analyze *discretely-valued^1^* time series data and is built to achieve two grounding objectives: computational efficiency and user flexibility. The first of these objectives is achieved by the core component of Inform, a high efficiency C library that takes care of the computation of information measures. The second objective is achieved through the design of a simple API and the development of a suite of wrappers for common higher-level programming languages, e.g., Python, R, Julia, and the Wolfram Language. The use of C as the implementation language and Inform’s carefully designed API make wrapping the core functionality straightforward. Since Inform has no external dependencies, distributing packages is greatly simplified. This is an advantage over libraries implemented in languages such as Java or R which require a virtual machine or an interpreter. Inform provides easy access to functions for empirically estimating probability distributions and uses them to compute common information-theoretic measures while also exposing a flexible API that a user can leverage to implement their own specialized measures. Additionally, Inform provides a collection of utilities that can be combined with other components of the framework to yield a wider range of analyses than those explicitly implemented. Inform provides a wide range of standard information-theoretic measures defined over time series and empirical probability distributions, as well as all of the common information dynamics measures. In addition, Inform provides a suite of functions for computing less common information-theoretic measures such as partial information decomposition (Williams and Beer, 2010), effective information (Hoel et al., 2013) and information flow (Ay and Polani, 2008). Inform v1.0.0 is released under the MIT license and is publicly available on GitHub^2^.

In this work, we introduce two of Inform’s language wrappers: PyInform^3^ (Python) and rinform^4^ (R). While the Inform library is, at least by C standards, straightforward to use, it is rather low-level. The decision to use C puts some of the memory-management burden on the user, and leads to rather rudimentary error handling. It is for these reasons that we invest the time in developing and maintain usable wrappers in a variety of higher-level languages. Without this initiative, users would have to call the C functions directly, decreasing the researcher’s productivity and cluttering their code. This is not to mention the error-prone nature of interfacing languages. By targeting some of the more common languages used in the field, we aim to make the software and algorithms accessible to a wide user-base. The language wrappers are designed to provide users with an experience that is idiomatic to their chosen language under the assumption that users will be more productive in a language with which they are familiar. Inform’s language wrappers are developed using the wrapping languages’ native technology, e.g., object-orientation in Python. This allows users to work with a programming interface written in their chosen language without requiring knowledge of the core C library but still benefiting from its implementation of optimized algorithms.

We begin with a review of the design and implementation of the Inform framework in Section 2. In Section 2.1 we describe the architecture of Inform and its wrappers with a focus on each of the four major components of the framework—distributions, information measures, time series measures and utilities. In Section 2.2 we discuss the validation process and stability of Inform, PyInform and rinform. In Section 3 we showcase the capabilities of the framework by analyzing three different collective systems: cellular-level biochemical processes in regenerating planaria (see Section 3.1), house-hunting behavior in Temnothorax ants (see Section 3.2), and consensus achievement in multi-agent simulations (see Section 3.3). Section 4 is dedicated to the analysis of the computational performance of Inform taking the JIDT library of (Lizier, 2014) as the reference framework and using active information and transfer entropy as benchmark metrics. Section 5 presents demonstrative examples of how to use PyInform and rinform with simple use cases for each of Inform v1.0.0’s major components. Finally, Section 6 concludes this paper with a discussion of the advantages and the shortcomings of the Inform framework as well as a summary of future directions of development.

##  2. Design and Implementation

Inform (MIT license)^5^ is a general-purpose library and framework for information-theoretic analysis of empirical time series data. Much of the design of Inform has focused on making the library (and its language wrappers) as intuitive and easy to use as possible, all the while attempting to provide powerful features that *some* other toolkits lack. Some of Inform’s features include:

- Optimized implementations of many common information-theoretic time series measures, including block entropy, mutual information, complete and apparent transfer entropy, active information storage and predictive information.
- Optimized implementations of less common concepts such as effective information, information flow, evidence for integration and partial information decomposition.
- All time series measures include local and average variants where applicable.
- An empirical probability distribution structure over a discrete event space^6^ and a suite of basic information-theoretic functions built around it.
- A collection of utility functions, such as black boxing and binning algorithms, which may be used in conjunction with time series measures to facilitate analysis of complex systems.
- No external library dependencies.

The Inform library is implemented in cross-platform C, and can be built on any system with a C11-compliant^7 ^compiler. The choice of C was not a simple one. The decision came down to two factors:

1. Essentially all modern programming languages provide a C foreign-function interface.
2. Most of Inform’s functionality requires minimal memory management — typically only one allocation and deallocation per function.
3. C does not have exceptions. While useful in a given language, exceptions make interfacing languages more difficult.
4. C requires no external dependencies for distribution — as such, the wrapper libraries do not depend on an external virtual machine, interpreter or JIT compiler.

All subsequent references to Inform will refer to the entire framework including its wrappers; any reference to the C library will be disambiguated as such.

###  2.1. Architecture

Information theory largely focuses on quantifying information within probability distributions. To model this, Inform is designed around the concept of an empirical probability distribution. These distributions are used to define functions which compute information theoretic quantities. From these basic building blocks, we implemented an entire host of time series measures. Intuitively, the time series measures construct empirical distributions and call the appropriate information-theoretic functions. These three components—distributions, information measures and time series measures—form Inform’s core functionality. Additionally, Inform provides a suite of utilities that can be used to augment and extend it’s core features. We now detail how these components are implemented and interact with each other to provide a cohesive toolkit.

Inform’s empirical probability distributions are implemented by a distribution class, Dist. This class, which is a wrapper for the C structure inform_dist, stores the relative frequencies of observed events that can then be used to estimate each event’s probability. The framework provides a suite of functions built around Dist which makes it easy for users to create distributions, accumulate observations and output probability estimates. It is important to note that Inform’s empirical distributions are only defined for discrete events. Subsequent releases will natively support continuous data (see Section 6).

Inform uses the Dist class to provide well-defined implementations of many Shannon information measures. In Python, the canonical example of such a function is

pyinform.shannon.entropy(dist, b = 2) 

which computes the (Shannon) entropy of the distribution dist using a base-b logarithm . Equivalently, the R function to compute Shannon entropy is given by

shannon_entropy(dist, b = 2)

Each measure in the framework takes some number of distributions and the logarithmic base as arguments, ensures that they are all valid^8^ , and returns the desired quantity. Inform v1.0.0 only provides information measures based on Shannon’s notion of entropy, but other types are planned for future releases (see Section 6).

Inform’s final core component is a suite of measures defined over time series. The version 1.0.0 release includes 15 time series measures with average and local (sometimes referred to as pointwise) variants provided where applicable. Each measure essentially performs some variation on the same basic procedure: first, accumulate observations from the time series into empirical distributions, and then, use them to compute some distribution-based information measure. Table 1 provides a complete list of the time series measures provided in Inform v1.0.0.

**Table 1:** The time series measures available in inform v1.0.0.

Time Series Measure	Local/Pointwise Variant
Block Entropy (Shannon, 1948)	✓
Cross Entropy (Cover and Thomas, 2005)	×*
(Multivariate) Mutual Information (Tononi et al., 1994; Cover and Thomas, 2005)	✓
Conditional Entropy (Cover and Thomas, 2005)	✓
Relative Entropy (Kullback and Leibler, 1951; Cover and Thomas, 2005)	✓
Entropy Rate (Cover and Thomas, 2005)	✓
Active Information (Lizier et al., 2012)	✓
Transfer Entropy (Schreiber, 2000; Kaiser and Schreiber, 2002; Lizier et al., 2008)	✓
Separable Information (Lizier et al., 2010)	✓
Predictive Information (Bialek et al., 2001a; Bialek et al., 2001b)	✓
Excess Information (Crutchfield and Feldman, 2003; Feldman and Crutchfield, 2003)	✓
Effective Information (Hoel et al., 2013; Hoel, 2017)	×
Information Flow (Ay and Polani, 2008)	×
Partial Information Decomposition (Williams and Beer, 2010)	×
Evidence of Integration (Biehl et al., 2016)	×

Local/Pointwise variants are implemented for all measures that reasonably admit them, signified by a ✓. A × denotes measures for which a local variant is not implemented.

* (×) Cross entropy’s local variant is equivalent to local block entropy, and is thus not implemented.

The final component of Inform is the utility suite. One of the greatest challenges of building a general-purpose framework is ensuring that it can be applied to problems that are outside of the authors’ initial use cases. Inform attempts to do this by first exposing the basic components of the library, distributions and information measures, and then providing utility functions that can be used to augment the core functionality. One particular example of this is the black_box^9^ function which losslessly produces a single time series from a collection of time series (see Section 5.4 for a detailed description and an example of use of this particularly versatile function). The black_box function allows Inform to avoid implementing multivariate variants of time series measures while still making it straightforward for users to compute such quantities. Of course, there are a multitude of uses for such a function. Our aim is that the utility suite can extend Inform’s functionality well beyond what the authors had in mind when implementing the core library.

###  2.2. Validation

The Inform framework was developed using a test-driven approach: unit tests were written for each component before implementing the component itself. Consequently, all features in Inform have been thoroughly unit tested to ensure that they perform as expected. In fact, the bulk of the development effort went into testing, and test code accounts for roughly 60% of the entire C source code distribution.

To ensure cross-platform support, continuous integration services are employed to build and run all unit tests on multiple platforms. Travis CI^10^ builds currently ensure support for Linux with the gcc 4.6.3 and clang 3.4 compilers, and Mac OS X with AppleClang 7.3.0.7030031. AppVeyor^11^ builds ensure support for Windows with Microsoft Visual Studio 14 2015. Code coverage reports for PyInform and rinform are hosted by CodeCov^12^ and currently show a coverage of 97% and 91%, respectively, while coverage for the C implementation is in the works for future releases.

##  3. Analysis of Collective Behaviors

In this section, we illustrate the use of Inform by performing information-theoretic analyses of three collective behaviors: the dynamics membrane potentials and ion concentrations in regenerating planaria, nest-site selection by colonies of the ant *Temnothorax rugatulus*, and collective decision-making in a multi-agent system. While the following results are interesting in their own right, and will likely be considered more deeply in subsequent work, our primary focus is on showcasing the utility and range of the Inform framework.

###  3.1. Biochemical Collectivity in Regenerating Planaria

In this first case study, we use partial information decomposition (Williams and Beer, 2010) to analyze how various ions contribute to the cell membrane potentials in a regenerating planarian. Planaria are an order of flatworms which have prodigious regenerative abilities (Sheĭman and Kreshchenko, 2015). When a planarian is cut in half, each piece will regenerate the missing tissue and develop into a fully functional individual. Recent work is stored in a complex biophysical circuit which is not hardwired by the genome (Oviedo et al., 2010; Beane et al., 2011; Emmons-Bell et al., 2015; Durant et al., 2017). Many pharmacological reagents that target the endogenous bioelectrical machinery (ion channels and electrical synapses known as gap junctions) can alter the behavior of this circuit and thus alter the large-scale bodyplan to which fragments regenerate. An example of this is ivermectin, a chloride channel opener, which results in the development of a two-headed phenotype upon regeneration (Beane et al., 2011). The resulting two-headed morphology is persistent under subsequent regeneration events outside of the presence of ivermectin. The hypothesis is that these gap-junction inhibitors disrupt proper bio-electric communication between cells and lead the organism to non-wildtype morphological attractors. As an initial step at understanding how the morphological information is stored and modified, we can look at how information about the bio-electric patterning is stored in specific intracellular ion concentrations of $Na^{+}$, $K^{+}$, $Ca^{2+}$ and $C\,l^{-}$.

**Figure 1:**
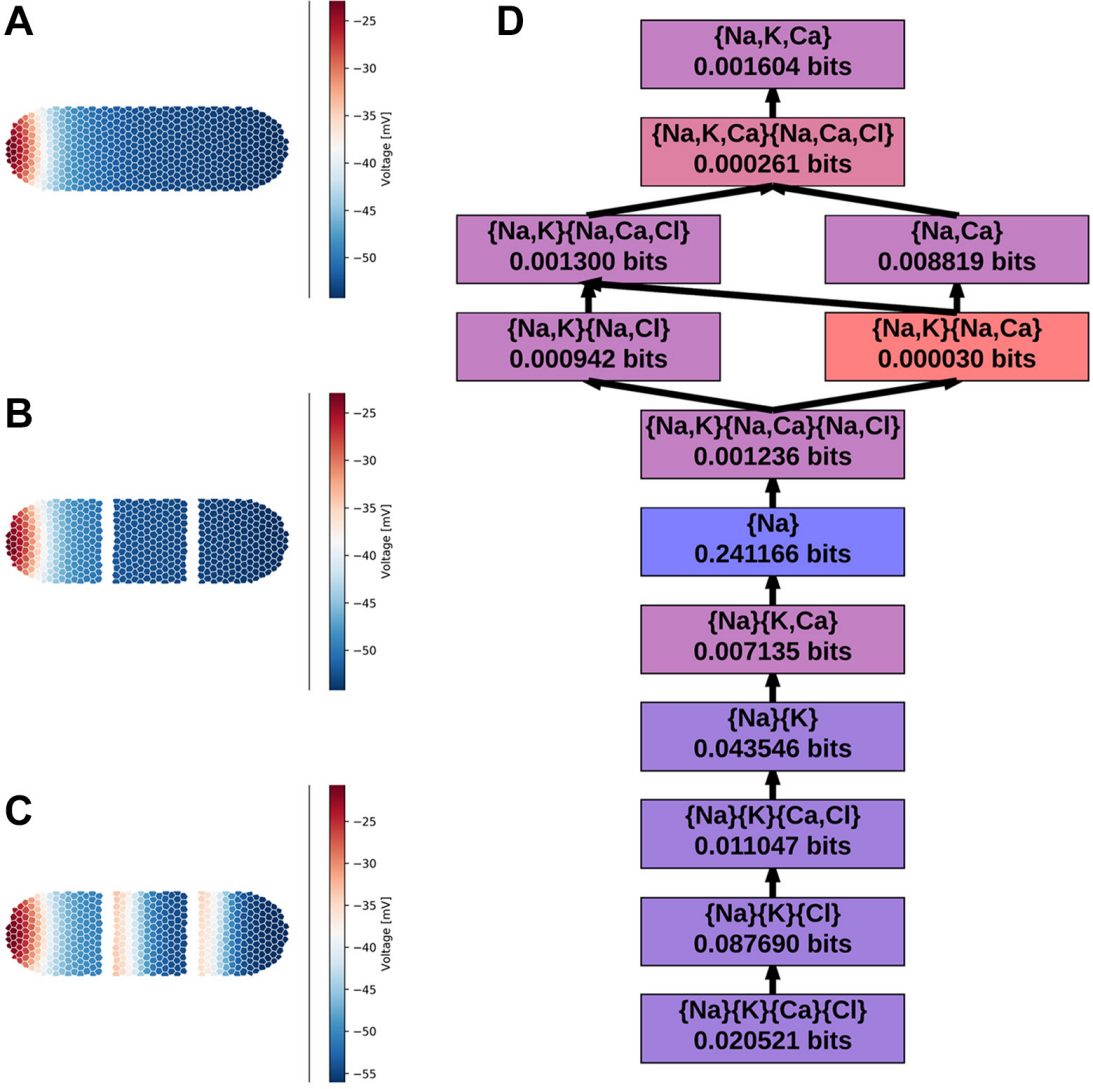
The $V_{m\,e\,m}$ distribution over the body of a BETSE simulated planarian (Pietak and Levin, 2016) over the course of regeneration: **(A)** pre-surgery, **(B)** 0s post-surgery, **(C)** 1000s post surgery. **(D)** The non-zero redundancy sub-lattice computed via partial information decomposition. Each node presents the redundant information provided by the given collection of random variables. Of the 166 nodes in the full redundancy lattice, these 13 are the only nodes which yield non-zero unique information. All other nodes were pruned, and the edges were constructed using the Williams-Beer dependency relations. Nodes are colored roughly by the order of magnitude of their unique information content.

We use the BioElectric Tissue Simulation Engine (BETSE) (Pietak and Levin, 2016) to simulate the planarian regeneration process under a simple two-cut intervention (Pietak and Levin, 2017). For this demonstrative case study, we simulate the planarian for 1000 s after two surgical cuts are made, dividing the worm into three pieces Figure 1A-C. From the simulation we extract the time series, sampled at a frequency of 10⁢H⁢z (10,000 time steps), of the average cell membrane potentials $V_{m\,e\,m}$ and the $Na^{+}$, $K^{+}$, $Ca^{2+}$ and $C\,l^{-}$ ion concentrations for each cell. We use a “threshold” binning to bin the average cell membrane potentials using a biologically realistic activation threshold of -40⁢m⁢V, the cell is considered depolarized (state 1) when $V_{m\,e\,m}$ is above -40⁢m⁢V, and hyperpolarized (state 0) otherwise. Each of the ion concentrations are separately binned into two uniform bins whose sizes depend on the range of the ion’s concentration.

From these binned data, we compute the partial information decomposition (PID) of the information about $V_{m\,e\,m}$ provided by the ion concentrations. From the 4 ion variables, Inform constructs the full 166-node redundancy lattice; however, only 13 of those nodes represent variable combinations that contribute unique information, in the sense of (Williams and Beer, 2010). We pruned all but those 13 variable combinations. The resulting sub-lattice is depicted in Figure 1D. Altogether, the intracellular ion concentrations yield approximately 0.425 bits of information about the average cell membrane potential – computed as the sum of the unique information provided by each node. This is less than the theoretical maximum of 1 bits, but that’s hardly surprising given that the cell membrane potential is determined by the difference between the intra- and extracellular ion concentrations. We also see that the only individual ion that provides any unique information about $V_{m\,e\,m}$ is $Na^{+}$ – $Na^{+}$ is the only ion that appears alone in Figure 1D. We know that both $Na^{+}$ and $K^{+}$ play a crucial role in determining $V_{m\,e\,m}$, so it is surprising to see that $Na^{+}$ is the dominate information provider. Subsequent work will delve deeper into the what this decomposition tells us about the biochemical mechanisms of regeneration.

As we conclude this example, it is worthwhile to acknowledge that Inform’s current implementation of PID is limited to Williams’s and Beer’s $I_{m\,i\,n}$ measure of redundant information (Williams and Beer, 2010). A number of alternative measures of redundancy and uniqueness could be applied to the redundancy lattice, e.g. (Bertschinger et al., 2014), and there is continuing discussion as to which is the “correct” measure. A subsequent version of PID will allow the user to specify which measure they would prefer, and even allow them to implement their own.

###  3.2. Nest-Site Selection by the Ant *Temnothorax Rugatulus*

In this case study, we use local active information to analyze collective decisions made by the ant *Temnothorax rugatulus* (Pratt et al., 2002; Sasaki et al., 2013). Specifically, we consider nest-site selection, a popular and well-studied collective behavior observed both in honeybee swarms and ant colonies (Franks et al., 2002). When Temnothorax ants need to choose a new nest, individuals in the colony explore the surrounding environment looking for possible candidate sites (e.g., a rock crevice). Upon the identification of a good candidate, an ant may perform a tandem run—a type of recruitment process whereby the ant returns to the old nest to lead another member of the colony in a tandem to the newly found site for a possible assessment. Tandem runs, together with independent discoveries of the same site, allow for a build up of a population of ants at that site which in turn triggers the achievement of a quorum, i.e., the identification by individual ants of the popularity of a candidate site. After quorum is reached, ants switch from performing tandem runs to performing transport—a type of recruitment process distinct from tandem runs whereby an ant returns to the old nest, loads another ant on her back and carries that ant to a site. The combination of parallel exploration, tandem runs, quorum sensing and transports allows Temnothorax ants to concurrently evaluate different candidate sites and converge on a collective decision for the best one.

For this study, we look at a live colony of 78 T. *rugatulus* ants repeatedly choosing between a good and a mediocre site in a laboratory environment for a total of 5 experiments. We consider ants to be in one of three state: uncommitted (state 0), committed to the good site (state 1) or committed to the mediocre site (state 2). All ants in the colony are individually paint-marked using a four-color code which allows us to identify individual ants and track their commitment state. From video-recordings of the experiments, we extract the commitment state of each ant over time as follows: initially, all ants are considered uncommitted, and ants commit to a certain site after performing a tandem run or a transport towards that site or when they are transported to that site. We record the commitment state of each ant every second and obtain 78 time series for each of the 5 experiments which we use to compute the local active information (history length k=2). As different experiments differ in duration due to the stochasticity inherent to colony emigrations, time series extracted from different experiments also differ in length (but all 78 time series within the same experiment have the same length). In our analysis, we considered shortened time series of $3\times 10^{4}$ time steps (approximately the same duration of the fastest emigration experiment) following a procedure described below.

**Figure 2:**
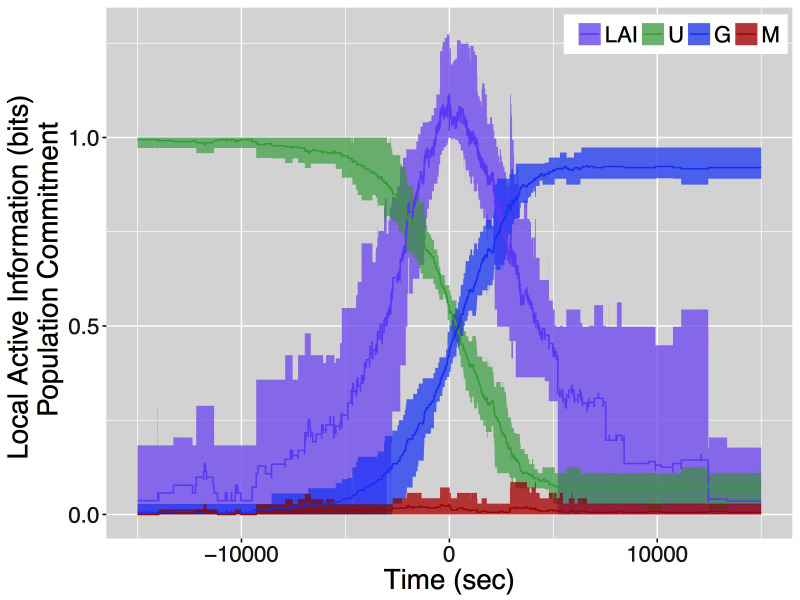
Distribution of local active information and colony-level commitment state for a live colony of 78 T. *rugatulus* ants computed over 5 colony emigrations. Lines represent mean values of local active information (**LAI**), and proportions of ants in the colony that are uncommitted (**U**), committed to the good site (**G**) and committed to the mediocre site (**M**). Shaded areas correspond to minimum and maximum values of the same quantities.

Figure 2 shows the results of our analysis of the local active information together with the change of commitment over time for the entire colony. Data are aggregated as follows: we first compute the mean local active information of individual ants in a colony emigration; then, we find the point in time where local active information peaks; finally, we center the local active information and the colony-level commitment state for each emigration around this point in time (i.e., time 0 in Figure 2) and compute mean, maximum and minimum values over experiments. The peak in the local active information is approximately in the middle of the decision-making process (i.e., when half of the colony is committed for the good site and half is still uncommitted). This maximum of the local active information, approximately 1 bit, identifies a critical point in the collective decision.

###  3.3. Multi-Agent Simulations

In this final case study, we use transfer entropy to analyze the flow of information in a multi-agent system developed to study the best-of-n problem (Valentini et al., 2017). Specifically, we consider a system where a collective of agents needs to chose between two options: 0 or1. The behavior of each agent is defined as a probabilistic finite-state machine with 2 states for each option: exploration and dissemination. In the exploration state, an agent explores the environment and evaluates the quality of its currently favored option. In the dissemination state, an agent promotes its opinion (i.e., broadcast its preference for a particular option to its neighbors) for a time proportional to the quality of its favored option. At the end of the dissemination state, soon before transitioning to the exploration state, the agent collects the preferences of its neighbors and applies a decision rule to reconsider its current preference. In this case study we consider two decision rules: the majority rule, whereby an agent adopts the option favored by the majority of its neighbors, and the voter model, whereby an agent adopts the option favored by a randomly chosen neighbor (Valentini et al., 2016).

We consider a collective of 100 agents tasked with a binary decision-making problem where the best option has quality 1.0 and the other option has quality 0.9. All the agents in the collective apply the same decision rule (i.e., either the majority rule or the voter model) over a neighborhood represented by the agent’s 5 nearest-neighbors. For each decision rule, we performed 1000 multi-agent simulations where the initial preferences of the agents are equally distributed among the two options. We let simulations run for a total duration each of $10^{4}$ seconds. Our aim is to use transfer entropy to analyze the flow of information to an agent from its neighborhood as it applies its decision rule. We extract a binary-state series of preferences for each agent, where each element of the series is the agent’s preference immediately prior to apply it’s decision rule. We then construct a 6-state series of neighborhood states, each element of which is the number of neighbors with a preference for the best option (i.e., {0,…,5}) at the time of the agent’s decision. As opposed to the previous case study, each simulation lasts for the same amount of time. However, the number of applications of a decision rule by an agent within the same simulation and across different simulations is stochastic. Consequently, time series derived from different agents differ in length (on average, 13.93±3.27 for the majority rule and 13.82±2.89 for the voter model). To mitigate the effect of short time series, we used time series from all agents within a simulation to compute the probability distributions required for transfer entropy (i.e., an average of 1393 samples for the majority rule and 1382 for the voter model) and consider this quantity an average over all agents of the collective. In this system, agents are memoryless and parameters have been tuned to approximate a well-mixed interaction pattern. However, time correlation may still be present as a result of the interaction of agents with their neighborhood. For simplicity, we use a history length of k=1 and let the investigation of longer history lengths for future work.

**Figure 3:**
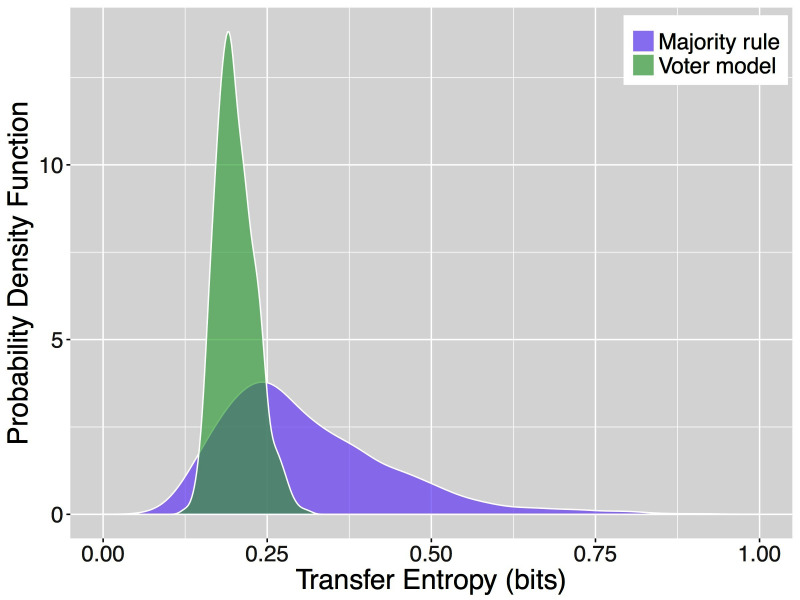
Probability density functions of the average transfer entropy for agents in systems applying the majority rule (purple) and for agents in systems using the voter model (green).

Figure 3 shows the results of our analyses of the multi-agent simulations. Specifically, it depicts the probability density functions (PDF) of the average transfer entropy toward an agent applying a decision rule over 1000 simulations. To compute the average transfer entropy towards an agent, we estimate the required probability distributions from the time series of all agents in the collective and use these distributions to obtain one sample of transfer entropy for each simulation. The PDFs of transfer entropy obtained for the majority rule and for the voter model are remarkably different (two sample t-test, p-value $<2.2\cdot 10^{-16}$). On average, the majority rule has a higher value of transfer entropy (0.3106 bits) with respect to the voter model (0.2019 bits). However, it is also characterized by a larger spread with a SD of 0.1302 bits compared to that of the voter model, 0.0301 bits. Previous analysis of these decision mechanisms under similar conditions showed that the majority rule is much faster than the voter model and its consensus time has an higher variance as well (Valentini et al., 2016). These results are likely correlated and a deeper analysis of this case study is currently undergoing.

##  4. Performance Analysis

In this section, we investigate the performance of PyInform by calculating two computationally demanding measures of information dynamics: active information (AI) and transfer entropy (TE). While we focus on PyInform here, rinform shows comparable performance characteristics. We compare the performance of PyInform with that of JIDT (Lizier, 2014) which we take as the gold-standard for the field. We chose AI and TE as they are the primary overlap in the functionality of PyInform and JIDT. The time series for the following tests were generated using the same multi-agent simulation described in Section 3.3. The state of each agent includes its opinion (i.e., 0 or 1) and its control state (i.e., dissemination or exploration). As such, the time series for each agent is base-4 and runs for the entire duration of the simulation, not just the decision points as in Section 3.3. We considered four different data sets wherein we varied both the decision rule (i.e., majority rule or voter model) and the difficulty of the decision-making problem (i.e., $\rho_{0}=1.0$ and $\rho_{1}\in \left\{0.5,0.9\right\}$). For each data set, we executed 1000 simulations with a duration of 1001 time steps using a collective of 50 agents initialized with an equal distribution of preferences for both options.

Using the four data sets described above, we computed the AI for each agent in the collective and the TE using PyInform and JIDT’s built-in time series-based functionality. We computed AI and TE for history lengths 1≤k≤11 or until computational resources were exhausted. For each data set and history length k, we repeated 5 times the calculations and timed the computational process. In computing the run times, we considered only the time necessary to loop over the agent combinations and to compute the relevant values while we disregarded the time spent reading data files and comparing results. All performance tests were single-threaded and run with Amazon Web Services, using a c4.large EC2 instance relying on a 2 vCPUs and 3.75 GB of RAM^13^

**Figure 4:**
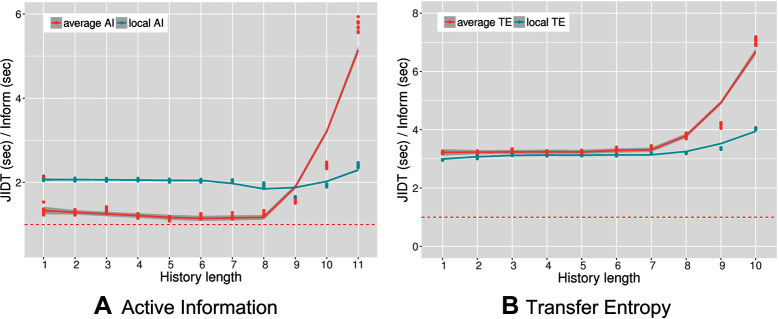
Performance ratio versus history length for average and local active information **(A)** and average and local transfer entropy **(B) **The dashed lines mark a performance ratio of 1.0. Memory constraints limited computation of transfer entropy with JIDT up to k=10.

Figure 4 shows the results of the performance comparison as the ratio of execution times between JIDT and PyInform for active information (left panel) and transfer entropy (right panel). In both experiments, the PyInform package outperforms JIDT with a speedup ranging from a minimum of 1.2× up to a maximum speedup of 7×. The computational gain of PyInform over JIDT is more pronounced when computing average measures with a history length k>8 both in the case of AI and in that of TE. It is obligatory to note that history lengths k>8 are rarely useful in practice as the amount of data necessary for the measures to show statistical significance grows exponentially in k. We include the longer history lengths, simply to acknowledge that both frameworks experience exponential growths in runtime as k grows. As one would expect, the computational requirements of transfer entropy are greater than those of active information for both frameworks.

In addition to comparing the runtime performance, we also compared the absolute results of the calculations for all values of k. The values computed with the PyInform package never differed from those of the JIDT library by more than $10^{-6}\,\text{bits}$. PyInform is *marginally* more computationally efficient than JIDT while providing equally accurate calculations of information-theoretic measures. However, it is important to remember that computational performance is not the only aspect that one should consider when choosing a software solution. Developer time is often more valuable than computation time. For example, JIDT offers many benefits over Inform including its support for continuously-valued data and a wider range of parameters (e.g., source embedding, embedding delays, source-target delay). Subsequent versions of Inform will reduce the discrepancy in features (see Section 6), and the library wrappers are designed to increase programmer productivity. Whether or not speed is a deciding factor in a user’s decision to use Inform will depend on the requirements of the task at hand.

##  5. Use Case Examples

In this section we provide a few examples of how to directly use the Python and R wrappers, respectively, PyInform and rinform. Live documentation of these wrappers can be found at https://elife-asu.github.io/PyInform and https://elife-asu.github.io/rinform.

###  5.1. Empirical Distributions

We start with a simple example of how to use the Dist class to estimate a probability distribution from a binary sequence of events (seeListing 1 for PyInform and Listing 2 for rinform). In Python, the from_data static method creates a distribution and records observations from an array of discrete events. The same objective can be achieved in R using the infer function. In this case, two observations are made of the event “0” and three of event “1”. The probability method can be used to query the estimated probability of a given event. Alternatively, the dump method can then be used to return an array of all estimated probabilities.

Listing 1 **Estimate a probability distribution from a binary sequence of events. (Python)**

 In [1]: from pyinform import Dist

 In [2]: dist = Dist.from_data([0,1,1,0,1]) *# observe 2 0’s and 3 1’s*

 In [3]: dist

 Out[3]: Dist.from_hist([2, 3])

 In [4]: dist.probability(0) *# What is the probability of seeing a 0?*

 Out[4]: 0.4

 In [5]: dist.probability(1) *# What is the probability of seeing a 1?*

 Out[5]: 0.6

 In [6]: dist.dump() *# output the probabilities to an array Out[6]:* array([0.4, 0.6])

Listing 2 **Estimate a probability distribution from a binary sequence of events. (R)**

 In [1]: library(rinform)

 In [2]: dist <- infer(c(0,1,1,0,1)) *# observe 2 0’s and 3 1’s*

 In [3]: dist

 Out[3]: $histogram: [1] 2 3

 Out[3]: $size: [1] 2

 Out[3]: $counts: [1] 5

 Out[3]: attr(,”class”): [1] ”Dist”

 In [4]: probability(dist, 1) *# What is the probability of seeing a 0?*

 Out[4]: 0.4

 In [5]: probability(dist, 2) *# What is the probability of seeing a 1?*

 Out[5]: 0.6

 In [6]: dump(dist) *# output the probabilities to an array*

 Out[6]: [1] 0.4 0.6

This is only a sample of the functionality provided around the Dist class. Further examples can be found in the live documentation of PyInform^14^ and rinform ^15^

###  5.2. Shannon Information Measures

As described in Section 2.1, the Shannon information measures are defined around the Dist class. In this subsection, we give an example of how to compute the Shannon entropy of a distribution. In Listing 3, we demonstrate how to construct a Dist instance and compute its entropy using PyInform while Listing 4 shows the equivalent implementation using rinform. The resulting distribution can record observations of two events, “0” or “1”. With the distribution in hand, the accumulate function accumulates the observations from an array. This is functionally equivalent to Dist.from_data which was used in Listing 1 (Python) and infer which was used in Listing 2 (R). Once the distribution has been created, computing its entropy is as simple as performing a single function call to shannon.entropy (in Python) or shannon_entropy (in R).

Listing 3 **Estimate the entropy of an empirical distribution of binary events. (Python)**

 In [1]: from pyinform import shannon

 In [2]: from pyinform import Dist

 In [3]: dist = Dist(2) *# create a Dist over two events*

 In [4]: dist.accumulate([0,1,1,0,1]) *# accumulate some observations*

 Out[4]: 5 *# 5 observations were made*

 In [5]: shannon.entropy(dist, b = 2) *# compute the base-2 Shannon entropy*

 Out[5]: 0.9709505944546686

Listing 4 **Estimate the entropy of an empirical distribution of binary events. (R)**

 In [1]: library(rinform)

 In [2]: dist <- Dist(2) *# create a Dist over two events*

 In [3]: dist <- accumulate(dist, c(0,1,1,0,1)) *# accumulate some observations*

 In [4]: shannon_entropy(dist, b = 2) *# compute the base-2 Shannon entropy*

 Out[5]: [1] 0.9709506

A host of information measures are provided in the Inform framework. These can be found in the pyinform.shannon module^16^ for PyInform. While rinform is not organized into modules, the user has access to all the same information measures as described in the rinform’s documentation^17^

###  5.3. Time Series Measures

The time series measures are a primary focus for the Inform framework. Listing 5 (Python) and Listing 6 (R) provide a complete example of how to estimate the average and local (pointwise) transfer entropy between two base-4 time series — this functionality was used in the performance analysis described in Section 4. To demonstrate this, we construct^18^ a source time series, src, and then shift and copy it to a target time series, target. The expected result is that the average transfer entropy from src to target will be near 2.0 bits. The transfer_entropy function is employed to compute this value. The examples go on to compute the local transfer entropy, which returns an array of local (pointwise) values.

Listing 5 **Estimate the average and local transfer entropy from discrete data. (Python)**

 In [1]: import numpy as np

 In [2]: from pyinform import transfer_entropy

 In [3]: np.random.seed(2018)

 In [4]: src = np.random.randint(0, 4, 100)

 In [5]: target = np.zeros(len(source), dtype = int)

 In [6]: target[1:] =src[:−1]

 In [7]: transfer_entropy(src, target, k = 1) *# TE with history length 1*

 Out[7]: 1.8705725949309469

 In [8]: lte = transfer_entropy(src, target, k = 1, local = True) *# Local TE*

 In [9]: lte.shape

 Out[9]: (1, 99)

 In [10]: np.mean(lte) *# the mean local TE is approximately the*

 Out[10]: 1.870572594930947 *# same as Out[7]*

 In [11]: lte = transfer_entropy(src, target, k = 0)

  … *# stack trace removed for brevity*

 InformError: an inform error occurred - ”history length is zero”

Time series measures can fail for a variety of reasons ranging from invalid arguments to exhausted system memory. In these situations, an error is raised which describes the reason for the function’s failure. At the end of both Listing 5 and Listing 6, we provide an example of an erroneous function invocation. Pyinform raises an InformError while rinform prints an error message.

Listing 6 **Estimate the average and local transfer entropy from discrete data. (R)**

 In [1]: library(rinform)

 In [2]: set.seed(2018)

 In [3]: src <- sample(0:3, 100, TRUE)

 In [4]: target <- c(src[100], src[1:99])

 In [5]: transfer_entropy(src, target, k = 1) *# TE with history length 1*

 Out[5]: [1] 1.912181

 In [6]: lte <- transfer_entropy(src, target, k = 1, local = TRUE) *# Local TE*

 In [7]: dim(lte)

 Out[7]: (99, 1)

 In [8]: mean(lte) *# the mean local TE is approximately the*

 Out[8]: [1] 1.912181 *# same as Out[5]*

 In [9]: lte <- transfer_entropy(src, target, k = 0)

 Out[9]: Error: <k > is less then 1!

All of the time series measures follow the same basic calling conventions as transfer_entropy. Further examples of the various time series measures can be found in the live documentation of PyInform^19^ and rinform ^20^

###  5.4. Utility Functions

Our next example, Listing 7 and Listing 8, demonstrates how to use Inform’s utility functions to estimate the multivariate active information of two continuous time series, node1 and node2. It begins by binning points in each time series into one of two bins, x<0.5 or x≥0.5, using the bin_series function. Once binned, the series are black-boxed, that is, their states are aggregated together over a larger state-space, using the black_box function to produce a base-4 time series (i.e., the product of the bases of node1 and node2). Each time step of this black-boxed time series, series, represents the joint state of the two binned time series. From series, the multivariate active information with k=1 is estimated using the active_info function.

Listing 7 **Estimate the average multivariate active information of two continuous time series. (Python)**

 In [1]: from pyinform import active_info

 In [2]: from pyinform.utils import bin_series, black_box

 In [4]: threshold = 0.5

 In [5]: node1, _, _ =bin_series([0.5, 0.2, 0.6, 0.8, 0.7], bounds = [threshold])

 In [6]: node1

 Out[6]: array([1, 0, 1, 1, 1], dtype = int32)

 In [7]: node2, _, _ =bin_series([0.1, 0.9, 0.4, 0.7, 0.4], bounds = [threshold])

 In [8]: node2

 Out[8]: array([0, 1, 0, 1, 0], dtype = int32)

 In [9]: series = black_box((node1, node2))

 In [10]: series

 Out[10]: array([2, 1, 2, 3, 2], dtype = int32)

 In [11]: active_info(series, k = 1)

 Out[11]: 1.

Listing 8 **Estimate the average multivariate active information of two continuous time series. (R)**

 In [1]: library(rinform)

 In [3]: threshold <- 0.5

 In [5]: node1 <- bin_series(c(0.5, 0.2, 0.6, 0.8, 0.7), bounds = threshold)$binned

 In [6]: node1

 Out[6]: [1] 1 0 1 1 1

 In [7]: node2 <- bin_series(c(0.1, 0.9, 0.4, 0.7, 0.4), bounds = threshold)$binned

 In [8]: node2

 Out[8]: [1] 0 1 0 1 0

 In [9]: series <- black_box(matrix(c(node1, node2), ncol = 2), l = 2)

 In [10]: series

 Out[10]: [1] 2 1 2 3 2

 In [11]: active_info(series, k = 1)

 Out[11]: [1] 1

The flexibility of the the black_box function makes it worthwhile to elaborate further on precisely what it does. In making concurrent observations of a collection of random variables, say $X_{1},X_{2},\ldots$, which may or may not be correlated with one another, we are in fact making observations of an underlying variable W defined over a different state space Ω. These observed variables can be thought of as views, filters or projections of the the underlying system state drawn from Ω. Many information analyses require the reconstruction of Ω from the observations of $X_{1},X_{2},\ldots$. The black_box function covers this role in Inform. Given a number of time series, each representing the time series of a random variable, black_box losslessly encodes the joint state of those time series as a single value in the system’s joint state space Ω. As a concrete example, consider the following time series of concurrent observations of two random variables

X: 0,1,1,0,1,0,0,1,

Y: 1,0,0,2,1,2,1,2.

Here, X is a binary variable while Y is a trinary one. Together, observations of X and Y may be thought to represent observations of an underlying state variable W=(X,Y)∈Ω^21^:

W:(0,1),(1,0),(1,0),(0,2),(1,1),(0,2),(0,1),(1,2).

As such, these observations can be encoded as a base-6 time series which is precisely what black_box does, yielding

W: 1,3,3,2,4,2,1,5.

The black_box function accepts a host of arguments which augment how it constructs the resulting time series, all of which are described and demonstrated in the documentation^22^.

Inform’s collection of utilities allows the user to easily construct new information-measures over time series data. Combining utility functions such as black_box with common time series measures such as mutual_info is a powerful way for the user to extend the functionality of the Inform framework to include measures of particular interest to their research.

We will now conclude this section with two demonstrative examples of how black_box can be combined with the time series functions block_entropy^23^ and mutual_info to implement *conditional entropy* and *active information*, respectively. First recall that the conditional entropy of a random variable X conditioned on a random variable Y is defined as

(1) $$H(X\;|\;Y)=-\sum_{x,y}p(x,y)\log p(x\;|\;y)=H(X,Y)-H(Y).$$

As such, one might compute the conditional entropy by first constructing the joint distribution (X,Y) (using black_box) and then computing the difference of entropies as in Equation (1) (using block_entropy). This is demonstrated using PyInform in Listing 9 and rinform in Listing 10.

Listing 9 **Estimate conditional entropy between two time series using black_box and block_entropy. (Python)**

 In [1]: from pyinform import block_entropy, conditional_entropy

 In [2]: from pyinform.utils import black_box

 In [3]: X = [0,1,2,2,2,2,0,1,0] *# the target variable*

 In [4]: Y = [0,0,1,1,1,1,0,0,0] *# the condition variable*

 In [5]: XY = black_box((X,Y)) *# the joint variable (X,Y)*

 In [6]: conditional_entropy(X, Y) *# H(X | Y) =H(X,Y) - H(Y)*

 Out[6]: 0.5394169969192604

 In [7]: block_entropy(XY, k = 1) - block_entropy(Y, k = 1)

 Out[7]: 0.5394169969192604

Listing 10 **Estimate conditional entropy between two time series using black_box and block_entropy. (R)**

 In [1]: library(rinform)

 In [2]: X <- c(0, 1, 2, 2, 2, 2, 0, 1, 0) *# the target variable*

 In [3]: Y <- c(0, 0, 1, 1, 1, 1, 0, 0, 0) *# the condition variable*

 In [4]: XY <- black_box(matrix(c(X, Y), ncol = 2), l = 2) *# the joint variable (X,Y)*

 In [5]: conditional_entropy(Y, X) *# H(X | Y) =H(X,Y) - H(Y)*

 Out[5]: 0.539417

 In [6]: block_entropy(XY, k = 1) - block_entropy(Y, k = 1)

 Out[6]: 0.539417

Finally, we will perform a similar process to estimate the active information of random variable X as defined by

(2) $$A_{k}(X)=\sum_{x^{+},x^{\left(k\right)}}p(x^{+},x^{\left(k\right)})log\frac{p(x^{+},x_{i}^{\left(k\right)})}{p(x^{+})p(x^{\left(k\right)})}=I(X^{+},X^{\left(k\right)})$$

where $X^{+}$ is the random variable representing the state of X in the next time step and $X^{\left(k\right)}$ is the present k-history of X. We can use black_box to construct the time series of k-histories, and mutual_info to compute the mutual information between $X^{+}$ and $X^{\left(k\right)}$ as in Equation (2). We demonstrate this using PyInform and rinform in Listing 11 and Listing 12, respectively.

Listing 11 **Estimate active information of a time series using black_box and mutual_info. (Python)**

 In [1]: from pyinform import active_info, mutual_info

 In [2]: from pyinform.utils import black_box

 In [3]: X = [0,0,1,1,1,1,0,0,0]

 In [4]: X2 = black_box(X, k = 2) *# the 2-histories of X*

 In [5]: active_info(X, k = 2)

 Out[5]: 0.3059584928680418

 In [6]: mutual_info(X[2:], X2[:−1]) *# align indices of X and X2*

 Out[6]: 0.3059584928680421

Listing 12 **Estimate active information of a time series using black_box and mutual_info. (R)**

 In [1]: library(rinform)

 In [3]: X <- c(0, 0, 1, 1, 1, 1, 0, 0, 0)

 In [4]: X2 <- black_box(X, l = 1, r = 2) *# the 2-histories of X*

 In [5]: active_info(X, k = 2)

 Out[5]: 0.3059585

 In [6]: mutual_info(matrix(c(X[3:9], X2[1:7]), ncol = 2))

 Out[6]: 0.3059585

##  6. Conclusion and Discussion

In this paper we introduced Inform v1.0.0, a flexible and computationally efficient framework to perform information-theoretic analysis of collective behaviors. Inform is a general-purpose, open-source, and cross-platform framework designed to be flexible and easy to use. It builds on a computationally efficient C library and an ecosystem of foreign language wrappers for Python, R, Julia, and the Wolfram Language. Inform gives the user access to a large set of functions to estimate information-theoretic measures from empirical discretely-valued time series. These include classic information-theoretic measures such as Shannon’s entropy and mutual information, information dynamics measures such as active information storage and transfer entropy, and information-based concepts conceived to investigate the causal architecture of collective systems. Inform’s low-level API is organized around the concepts of probability distributions, information measures, time series measures and utilities and its flexibility allows users to construct new measures and algorithms of their own. We showcased the Inform framework by applying it to the study of three collective behaviors: cellular-level biochemical processes in regenerating planaria, colony emigration by the ant *Temnothorax rugatulus*, and collective decision-making in multi-agent simulations. We investigated the performance of the Inform framework by comparing them with those of the JIDT library showing that Inform have similar or superior performance with respect to JIDT. In effect, Inform is a potentially invaluable tool for any researcher performing information analysis of collective behaviors and other complex systems.

The Inform framework is still a relatively young project compared to more mature projects such as JIDT. While it has many features that make it unique such as, its computational efficiency, the large set of information-theoretic methods, and the availability of foreign language wrappers, it does lack some important functionality. We are planning three subsequent releases to incrementally extend the Inform framework. In the version 1.1.0 release, we will modify Inform’s interface to provide the user with access to the probability distributions used in the computation of information dynamics measures and their accumulation functions. In Python, for example, the extended API for computing the active information may take the following form:

class ActiveInfoAccumulator(Accumulator):

  def __init__(self):

   pass

  def accumulate(self, data):

   pass

  def evaluate(self, local = False):

   pass

The advantage of exposing probability distributions and their accumulation functions is that the user can modify the way that probabilities are estimated. As opposed to the version 1.0.0 where Inform’s time series measures require that all time series be stored in memory prior to the estimation of distributions, this new release will allow the user to write their own accumulation functions which could incrementally update distributions from very large time series stored on the hard-drive or with data that is generated in real-time. In the version 1.2.0 release, we will provide support for non-Shannon entropy functions. Shannon’s entropy of a discrete random variable is the unique functional form of entropy that satisfies all Shannon’s four axioms (Shannon, 1948). However, many functional forms of entropy become possible as soon as these four axioms are relaxed or otherwise modified. Two examples of such non-Shannon entropy forms are Rényi entropy (Rényi, 1961) and Tsallis-Havrda-Charvát entropy (Havrda and Charvát, 1967; Tsallis, 1988). Shannon’s entropy is currently used in the calculations of most information dynamics measures available in Inform. The version 1.2.0 release will allow the user to make use of Non-Shannon entropy functions which may give insight into the dynamics of information processing in non-ergodic systems. Finally, the version 2.0.0 release will represent a major improvement of the Inform framework by providing support for continuously-valued time series. Although Inform provides utilities to discretize continuous data through the process of binning, its repertoire of information-theoretic measures only supports discretely-valued time series. Discretely-valued time series allows for computational efficiency (complexity is O⁢(N) in the length of the time series N), however, the discretization of continuous data might introduce artifacts and reduce the accuracy of the overall analysis. In the version 2.0.0 release we will implement estimation techniques for continuous probability distributions, such as kernel density estimation (Rosenblatt, 1956; Parzen, 1962; Schreiber, 2000; Kaiser and Schreiber, 2002), with the aim of extending Inform’s reach towards continuously-valued data. More advanced estimation techniques, such as Kraskov-Stögbauer-Grassberger estimation (Kraskov et al., 2004), are planned for subsequent releases once we have a standardized API support of continuous data. Some additional details concerning future releases of the Inform framework are described on the Issues page^24^ of the GitHub repository where users are encouraged to suggest features or report bugs.

## Author Contributions

DGM designed and implemented the Inform library as well as the Python, Julia, and Mathematica wrappers. GV designed and implemented the R wrapper. All authors contributed to the conceptualization of the framework and to the writing of the manuscript.

## Conflict of Interest Statement

The authors declare that the research was conducted in the absence of any commercial or financial relationships that could be construed as a potential conflict of interest.

The reviewer, JL, declared a past collaboration with one of the authors, SW, to the handling Editor.

## Footnotes

1. While the current release of Inform only supports analysis of discrete time series, full support for continuous data is planned, see Section 6.

2. https://github.com/elife-asu/inform

3. https://github.com/elife-asu/pyinform

4. https://github.com/elife-asu/rinform

5. https://github.com/elife-asu/inform

6. Support for continuous event spaces is planned for v2.0.0, Section 6.

7. ISO/IEC 9899:2011: https://www.iso.org/standard/57853.html

8. An empirical distribution is considered invalid if it has no recorded events.

9. The naming of this function is intended to bring to mind the process of “black boxing” nodes in a network. That is, this function models drawing an opaque box around a collection of nodes, treating them as one unit with no known internal structure.

10. https://travis-ci.org/ELIFE-ASU/Inform, https://travis-ci.org/ELIFE-ASU/PyInform , https://travis-ci.org/ELIFE-ASU/rinform

11. https://ci.appveyor.com/project/dglmoore/inform-vx977 , https://ci.appveyor.com/project/dglmoore/pyinform , https://ci.appveyor.com/project/gvalentini85/rinform

12. https://codecov.io/gh/ELIFE-ASU/PyInform , https://codecov.io/gh/ELIFE-ASU/rinform

13. See https://aws.amazon.com/ec2/instance-types/ for the specifications of the c4.large EC2 instance..

14. http://elife-asu.github.io/PyInform/dist.html

15. https://elife-asu.github.io/rinform/#2_empirical_distributions.

16. http://elife-asu.github.io/PyInform/shannon.html

17. https://elife-asu.github.io/rinform/#3_shannon_information_measures

18. In Python, we use —numpy—, a package that provides a wealth of useful array-based functionality: http://www.numpy.org/.

19. http://elife-asu.github.io/PyInform/timeseries.html

20. https://elife-asu.github.io/rinform/#4_time_series_measures.

21. Note that if we had considered W′ = (Y,X) ϵ Ω′ instead, the encoded time series would have been different , e.g., 2,1,1,4,3,4,2,5. However, the mutual information between them, I(W,W′), tends to the theoretical maximum H(W) as the number of observations increases; this indicates that (X,Y) and (Y,X) are informationally equivalent representations of the underlying space.

22. http://elife-asu.github.io/PyInform/utils.html , https://elife-asu.github.io/rinform/#5_utilities.

23. The —block_entropy— function computes the Shannon block entropy of a time series. This reduces to the standard Shannon entropy when a block size of k = 1 is used, e.g., —block_entropy(series, k = 1)—.

24. https://github.com/elife-asu/inform/issues
